# Clinical presentation differences of lichen sclerosus in pre- and post-menopausal women

**DOI:** 10.61622/rbgo/2026rbgo15

**Published:** 2026-04-16

**Authors:** Fernanda Villar Fonseca, Gabriela Gracia Malinoski, Giorgia Flexa Zambon, Glória Maria Nassar, Helena Maria Prado Domingues, Heloisa Lipski Landarim, Leonardo Grazziolli

**Affiliations:** 1 Erasto Gaertner Hospital Department of Cervical Pathology Curitiba PR Brazil Department of Cervical Pathology, Erasto Gaertner Hospital, Curitiba, PR, Brazil.; 2 Universidade Positivo Medical School Universidade Positivo Curitiba PR Brazil Universidade Positivo Medical School, Universidade Positivo, Curitiba, PR, Brazil.

**Keywords:** Lichen sclerosus et atrophicus, Menopause, Premenopause, Vulvar disease

## Abstract

**Objective:**

To evaluate the clinical differences in the presentation of vulvar lichen sclerosus in pre- and postmenopausal women, aiming to provide insights to improve disease management and promote the health and well-being of affected patients.

**Methods:**

This was a retrospective analytical study involving 287 women diagnosed with vulvar lichen sclerosus, conducted between January 2009 and December 2023 at Erasto Gaertner Hospital, in Curitiba, Brazil. Clinical, epidemiological, and therapeutic management aspects were assessed and compared between pre- and postmenopausal patients.

**Results:**

Most cases (87%) occurred in women over 50 years of age, showing a strong association with menopause. Systemic arterial hypertension and type II diabetes mellitus were the most frequent comorbidities. Complaints of vulvar atrophy and anatomical deformities predominated among women over 50 years. Pruritus was the most common symptom in both groups, while dyspareunia was more prevalent among younger women. High-potency topical corticosteroid therapy was effective in more than half of the patients in both groups. Additionally, contrary to previous literature, 2% testosterone cream showed significant clinical improvement in the younger group. Progression to in situ neoplasia was observed in 11 cases, and progression to invasive neoplasia occurred in 6 cases.

**Conclusion:**

Differences in clinical presentation according to hormonal status reinforce the importance of individualized management strategies. Further studies with larger samples are necessary to optimize treatment approaches and improve quality of life for women affected by this chronic, mutilating, and often neglected dermatosis.

## Introduction

Lichen sclerosus (LS) is a chronic, autoimmune, inflammatory dermatosis that can occur in women of all ages, with a higher prevalence after menopause.^(1)^ Although this disease can affect the entire body, it is most commonly found in the vulvar and anogenital regions.^(2)^ LS presents with varied symptoms, the main ones being hypopigmentation and skin atrophy. Depending on the degree of lesion involvement, it may cause anatomical alterations in the genital region and progression to cancer.^(1)^ Importantly, LS can severely compromise patients’ quality of life.^(3,4)^

LS can occur at any age and in both sexes, with no racial predilection.^(1)^ In the general population, its prevalence ranges from 1:300 to 1:1,000 individuals.^(5)^ The female-to-male ratio is between 3:1 and 10:1.^(6)^ However, the true prevalence is underestimated and remains unknown, as the disease is often underdiagnosed and many patients are asymptomatic. There are two incidence peaks in women: the first between ages 8 and 13, and the second between the fifth and sixth decades of life, with the mean age at diagnosis being between 52 and 60 years.^(1)^

The pathogenesis of LS is not yet fully understood. It is known to have a multifactorial etiology, with genetic and epigenetic components, lymphocyte-mediated immune mechanisms, fibroblast proliferation, and oxidative stress all involved in disease development.^(7)^

The most characteristic symptoms of LS in women are hypopigmentation, vulvar fissures, labial resorption, and skin atrophy.^(8,9)^ Other important symptoms include sexual dysfunction, dyspareunia, pruritus, erythema, and skin sclerosis. Diagnosis is confirmed by skin biopsy, and treatment should be initiated as early as possible.^(10,11)^

The main risk factor for LS is trauma and chronic irritation. Pruritus, friction, and surgical procedures can trigger the disease, a phenomenon known as Koebner's response. In elderly women, urinary incontinence, multiparity, and poor genital hygiene are also risk factors. Additionally, there is a strong association between the use of immunotherapy and checkpoint inhibitors and the development of LS in cancer patients.^(7)^

It is important to emphasize that genital LS may progress to a type of cancer known as vulvar squamous cell carcinoma, observed in 3.5% to 7% of patients. Approximately 65% of vulvar carcinoma cases have a prior history of LS. The mechanism of this malignant transformation remains unknown.^(7)^

Therefore, this study aims to analyze the symptomatic differences of LS between pre- and postmenopausal women, providing clinical insights to improve early diagnosis and disease management. As clinical manifestations may vary according to life stage, investigating these differences is essential for guiding more accurate diagnoses and defining more effective treatments, thereby promoting health and well-being in patients.

Although the literature discusses prevalence, risk factors, and the association of LS with neoplasms, there remains a scarcity of studies directly comparing symptomatology between pre- and postmenopausal women. This gap is relevant because hormonal and anatomical changes related to climacteric may modify the clinical presentation of the disease, with direct implications for early diagnosis and therapeutic response. Thus, this study contributes to expanding scientific knowledge on LS and the clinical management of this condition in pre- and postmenopausal patients.

## Methods

This is a retrospective analytical study aimed at analyzing data from electronic medical records of women diagnosed with Lichen Sclerosus (LS). The research setting was Erasto Gaertner Hospital, located in Curitiba, Brazil, where patients were treated between January 2009 and December 2023.

Data collection was carried out by the researchers through the review of electronic records of 287 patients diagnosed with LS, using the hospital's specific digital system. Patients were identified only by medical record codes, ensuring confidentiality.

Women of all ages with a biopsy-confirmed diagnosis performed at the institution during the study period were included. Medical records with missing, inadequate, or incomplete information necessary for variable analysis were excluded.

To ensure confidentiality, patients were identified only by record codes, and their names were not tabulated. All data were stored in a restricted-access environment available only to the researchers, ensuring data protection and privacy.

The collected information included: age, menopausal status and duration, comorbidities, presence of other gynecological conditions, date of disease diagnosis, symptomatology, duration of symptoms, first proposed treatment, corticosteroid side effects, response to initial treatment, time to improvement, number of treatment modalities proposed, most effective treatments for lichen sclerosus, fissures and pruritus, need for surgical intervention, treatment adherence, length of clinical follow-up, and disease progression to vulvar intraepithelial neoplasia or invasive vulvar neoplasia.

The results were analyzed using both quantitative and qualitative variables. Associations between variables were assessed using appropriate statistical tests, such as chi-square and Fisher's exact test, with p-values < 0.05 considered statistically significant. Analyses were performed with SPSS software, version 19.

Prior to data collection, a literature review was conducted in the Scielo and PubMed databases using the descriptor "Lichen Sclerosus et Atrophicus." The review, in both English and Portuguese, guided the preparation of the research project, which was submitted to and approved by the Research Ethics Committee (CEP), under approval number 4.076.615, CAAE 30926420.0.0000.0098. Since this was a retrospective chart review, informed consent was waived. After approval, hospital data collection was initiated.

## Results

In this study, patients were divided into two age groups: women aged 21 to 50 years and women aged 51 to 96 years. The younger group accounted for 13% of patients (37 out of 287), while 87% (250 out of 287) were in the older group. Overall, 88% of the sample (252 patients) were in the climacteric period, while 12% (35 patients) were in the fertile period. From the analysis of first consultation records, pruritus was identified as the most prevalent symptom in both age groups, with similar percentages, affecting approximately 95% of both groups, as shown in table 1. Dyspareunia was reported in 19% of patients aged ≤50 years, approximately three times more frequent than in patients over 50 years (6.8%), with a statistically significant difference (p = 0.011; OR 3.19; 95% CI 1.22–8.34). Women under 50 years also reported multiple concomitant symptoms at diagnosis more frequently than older women (59% vs. 31%; p = 0.001), suggesting a greater impact on quality of life (Table 1). Vulvar pain and fissures were more common in younger women, although without statistical significance (Table 1). The interval between symptom onset and diagnosis with appropriate treatment ranged from 1 to 60 months in the younger group and from 1 to 120 months in the older group.

**Table 1:** Prevalence of symptoms in the first consultation by age group

Symptoms at the first visit	Less than or equal to 50 years old (21-50 years old = 37) n%	Over 50 years old (51-96 years old = 250) n%	p (IC 95%)	OR
Itchy	35 (95)	238 (95)	0.87 (0.18-4.10)	0.88
Atrophy	12 (32)	116 (46)	0.11 (0.26-1.15)	0.55
Vulvar pain	3 (8)	17 (6.8)	0.77 (0.33-4.34)	1.20
Dyspareunia	7 (19)	17 (6.8)	0.011 (1.22-8.34)	3.19
Cracks	5 (13)	29 (11)	0.73 (0.42-3.2)	1.19
Sexual dysfunction	0 (0)	0 (0)	-	-
Anatomical deformity	15 (40)	114 (45)	0.56 (0.4-1.64)	0.81
Associated symptoms	23 (59)	77 (31)	0.001 (1.62-6.6)	3.29

The main comorbidities associated with the condition presented by the patients were systemic arterial hypertension (SAH), type 2 diabetes mellitus (T2DM), hypothyroidism, and anxiety/depression. In addition, vitiligo and psoriasis were present in 2 patients (0.69% of the total). The comorbidity rate was substantially higher in the group over 50 years of age, mainly regarding SAH and T2DM. The higher prevalence of hypothyroidism in patients over 50 years (OR = 0.36; p > 0.05; 95% CI 0.083–1.58) and the higher percentage of anxiety/depression in younger patients (OR = 1.29; p = 0.69; 95% CI 0.35–4.66) were not statistically significant (Table 2).

**Table 2:** Distribution of comorbidities by age group

Most frequent comorbidities	Less than or equal to 50 years old (21-50 years old = 37) n%	Over 50 years old (51-96 years old= 250) n%
Hypothyroidism	2 (5)	34 (13)
Type 2 diabetes	1 (3)	47 (19)
Systemic arterial hypertension	4 (11)	87 (35)
Anxiety/depression	3 (8)	16 (6)
Vitiligo	0 (0)	1 (0.4)
Psoriasis	0 (0)	1 (0.4)

As with the higher prevalence of other associated diseases, the rate of progression to vulvar intraepithelial neoplasia (VIN) and invasive vulvar neoplasia was greater in women over 50 years of age, as shown in table 3. In addition to age, it was observed that progression to neoplasia occurred even in women who demonstrated adequate adherence to the proposed treatments and remained under follow-up with the healthcare team for variable periods, ranging from 6 to 42 months in patients with VIN and from 3 to 22 months in those with invasive vulvar neoplasia. The reason for discontinuation of follow-up was not assessed in this study. At the time of the diagnosis of VIN, only 9% (1/11) were undergoing therapeutic follow-up; this patient had temporarily discontinued corticosteroid use due to other comorbidities. Furthermore, 33% (2/6) of patients diagnosed with invasive disease had corticosteroid therapy replaced by other treatment modalities.

**Table 3:** Rate of progression to neoplasia by age group

Rate of progression to neoplasia	Less than or equal to 50 years old (21-50 years old) n%	Over 50 years old (51 - 96 years old) n%	Total
VIN	0/0 (0)	11/11 (100)	11/287 (4)
Invasive neoplasia	1/6 (16)	5/6 (84)	6/287 (2)

VIN: vulvar intraepithelial neoplasia

The initial treatment proposed for patients with LS varied according to age group and clinical presentation, as shown in table 4. Overall, primary approaches were proportionally similar when comparing the two groups, with high-potency corticosteroids being the most prevalent option. The combination of high-potency corticosteroids with sedative antihistamines, topical estrogen, and calcineurin inhibitors followed the same proportional pattern between the two groups, each with its respective percentage.

**Table 4:** First treatment proposed by age group

Proposed treatment	Less than or equal to 50 years old (21-50 years old = 37) n%	Over 50 years old (51-96 years old = 250) n%
High-potency corticosteroid	22 (60)	138 (55)
Association of high-potency corticosteroids + sedative antihistamines	9 (24)	60 (24)
High potency corticosteroid + topical estrogen association	2 (5)	23 (9)
Topical estrogen	3 (8)	19 (8)
Topical testosterone	0 (0)	1 (0.4)
Calcineurin inibitors	0 (0)	0 (0)
Others*	1 (3)	9 (3.6)

Others: skin barrier agents, emollients, and topical anesthetics

Regarding adverse effects related to treatment with high-potency corticosteroids, only 7.7% of patients (22/287) experienced undesirable reactions—8% in the ≤50-year group (3/37) and 7.6% in the >50-year group (19/250). Reported symptoms included burning sensation, dryness, irritation, fissures, and hypopigmentation, as well as skin atrophy, the latter considered the most severe event and observed in only two patients. These findings were classified as treatment-related side effects rather than lack of clinical response.

When therapeutic response itself was evaluated, 8% of the sample did not achieve complete remission after the initial approach. This condition was considered true therapeutic failure and was defined as the absence of significant clinical improvement despite regular and appropriate use of high-potency corticosteroids as prescribed. Treatment failure occurred in 11% of women ≤50 years and in 7.6% of women >50 years, with no statistically significant difference between the groups (p = 0.5; OR 1.47; 95% CI 0.47–4.59).

The results of treatments with the best clinical response are shown in (Table 5) and demonstrated that high-potency corticosteroids were effective in 51% of women aged ≤50 years (19 patients) and in 53% of women over 50 years (133 patients).

**Table 5:** Treatments with the best clinical response by age group

Treatment with best clinical response	Less than or equal to 50 years old (21-50 years old = 37) n%	Over 50 years old (51-96 years old = 250) n%
High-potency corticosteroid	19 (51)	133 (53)
Association of high-potency corticosteroids + sedative antihistamines	10 (27)	49 (20)
High potency corticosteroid + topical estrogen association	4 (11)	50 (20)
Topical estrogen	1 (3)	14 (5.6)
Topical testosterone	3 (8)	4 (1.4)
Calcineurin inibitors	0 (0)	0 (0)

Testosterone 2% cream (testosterone propionate 2%) was used as an adjuvant to high-potency corticosteroids in women with persistent complaints of dyspareunia and refractory fissures for an additional period of 2 to 3 months after partial response to the initial treatment. This therapeutic association showed a positive clinical response in 8% of women ≤50 years (3/37) and in 1.4% of women >50 years (4/250), conferring an approximately fivefold greater chance of complete symptom improvement in the younger group compared with the older group (p = 0.03; 95% CI 1.16–25.29; OR 5.42). These findings suggest that testosterone may represent a promising complementary alternative for younger patients with sexually impactful symptoms, such as dyspareunia, which are more prevalent in this age group. However, the small number of patients treated with this modality resulted in a wide confidence interval, reinforcing the need for larger studies to confirm the potential benefit of adjuvant testosterone therapy.

In addition, alternatives such as the combination of high-potency corticosteroids with antihistamines, topical estrogen, and the combination of topical estrogen with high-potency corticosteroids also proved effective, with variable percentages depending on the group analyzed.

## Discussion

The present study was based on the investigation of symptomatic differences of Lichen Sclerosus (LS) in pre- and postmenopausal women, with the primary aim of assessing how these differences may support and influence clinical practice. The final data analysis identified some significant differences among the studied factors, as well as similar patterns, both of which may impact the diagnosis and treatment of this condition.

Among the groups studied, there was a clear predominance of the disease in patients over 50 years of age, with 87% of cases occurring in women aged 51 to 96 years. This prevalence may be correlated with the fact that 88% of these patients were in the climacteric period. This finding is consistent with the existing literature, which highlights climacteric age as the period in which most diagnoses of LS are made.^(12)^

The analysis of comorbidities revealed a higher prevalence of hypertension, type 2 diabetes, and hypothyroidism among patients over 50 years, a result that is in line with the epidemiology of these conditions.^(13)^ These comorbidities, when associated with LS, may have a substantial impact on patients’ quality of life.^(3)^

Pruritus was the most prevalent symptom in both groups, being highly present in pre- and postmenopausal women. This finding is consistent with previous studies, which report pruritus as one of the most common symptoms of LS.^(8)^ Furthermore, it can be inferred that this symptom is common regardless of the woman's life stage, as no statistically significant difference was observed between the groups.

Atrophy and anatomical deformities were more frequent in patients over 50 years, most of whom were postmenopausal, although this result was not statistically significant. No differences were observed between groups regarding vulvar pain and sexual dysfunction, which may be related to the clinical setting, as patients were treated in an oncological hospital where these symptoms may have received less emphasis. However, dyspareunia was significantly more frequent in women under 50 years, and the presence of multiple concomitant symptoms at diagnosis was also more common in younger women, indicating a greater impact on quality of life when LS manifests earlier in life.^(4)^

The risk of presenting with dyspareunia or associated symptoms as the initial complaint was three times higher in patients under 50 years, which also represents a significant negative impact on quality of life.^(3)^ Furthermore, this finding may be related to the social context of the patients, reflecting a population that is potentially more sexually active, which can influence both sexuality and self-esteem.

Following the assessment of initial treatment, high-potency corticosteroids demonstrated the best clinical response in both groups, reinforcing their role as the first-line therapy for LS.^(1,2,7,12)^

Analysis of the results from this study's sample also showed that, although considered obsolete in the literature,^(10,14)^ 2% testosterone cream may be a promising complementary alternative to corticosteroids in younger women, particularly those with complaints of dyspareunia.^(3,8)^ These patients had a fivefold higher likelihood of achieving complete symptom resolution compared to women over 50 years.

However, the wide confidence interval and small sample size indicate the need for studies with larger cohorts to confirm this finding.

The rate of progression to neoplasia in this study showed that 4% of women (11 patients) developed Vulvar Intraepithelial Neoplasia (VIN). Notably, this progression occurred exclusively in women over 50 years, suggesting that advanced age may be a significant risk factor for the development of VIN.

Additionally, 2% of women (6 patients) progressed to invasive vulvar neoplasia. Among these cases, 16% occurred in women under 50 years (1 of 6 patients), while 84% occurred in women over 50 years (5 of 6 patients).

These findings suggest that, although progression to invasive neoplasia is less common, it also occurs more frequently in older women. This highlights the importance of rigorous and continuous follow-up, particularly for patients over 50 years, to enable early detection of neoplastic progression and even its prevention through appropriate treatment.^(7,15)^

It should be noted that the histopathological subtype of vulvar intraepithelial neoplasia was not evaluated in this study, as this information was not systematically available in the medical records reviewed. Therefore, the analysis was limited to identifying the presence of vulvar intraepithelial neoplasia or invasive vulvar neoplasia, and stratification of cases according to histological subtype was not possible. Consequently, early identification and appropriate management of these patients are crucial to improve clinical outcomes and reduce morbidity associated with vulvar neoplasia.

Furthermore, dyspareunia was confirmed as the most prevalent symptom among younger women, emphasizing its clinical significance. In this context, 2% testosterone cream appears as a valuable adjunct therapy to corticosteroids, demonstrating notable potential in symptom relief and improvement of patient quality of life. Another important finding is the relationship between age and the progression of LS to neoplasia, indicating a higher risk in postmenopausal women.

These results reinforce the importance of continuous and targeted follow-up for the prevention and early detection of complications. Overall, this study contributes to the scientific understanding of LS, demonstrating significant clinical differences between pre- and postmenopausal women, and supports the need to individualize clinical management according to life stage, guiding more personalized decisions with positive impact on both disease control and patient quality of life.

## Conclusion

The study reveals clinically relevant differences between pre- and postmenopausal women, particularly the higher frequency of dyspareunia and multiple symptoms in women under 50 years of age and the greater risk of neoplastic progression in postmenopausal women, reinforcing the need for individualized follow-up according to hormonal status. The results show that pruritus is the most frequent symptom in both groups, whereas dyspareunia and the presence of multiple concomitant symptoms were three times more common in younger women, indicating a greater impact on quality of life in this age group. In contrast, atrophy and anatomical deformities were more prevalent in women over 50 years. Treatment with high-potency corticosteroids demonstrated consistent efficacy across all ages, with a low rate of complications. Topical 2% testosterone cream showed favorable clinical response in younger women and may be considered an adjunctive option to corticosteroids, particularly for those with complaints of dyspareunia. This study emphasizes the importance of an individualized approach to LS management, considering epidemiological aspects and symptomatic differences at each stage of life. The limited number of younger women included in this research, as well as the lack of significant data on premenopausal patients, highlights the need for further studies with larger samples. Expanding research in this area will help refine diagnostic and treatment strategies, ultimately promoting better quality of life for women affected by this chronic, potentially mutilating, and frequently neglected condition in clinical practice.

## Data Availability

The research data are described in the article presented.
